# Perinatal HCV Transmission Rate in HIV/HCV Coinfected women with access to ART in Madrid, Spain

**DOI:** 10.1371/journal.pone.0230109

**Published:** 2020-04-09

**Authors:** Sara Domínguez-Rodríguez, Luis Prieto, Carolina Fernández McPhee, Marta Illán-Ramos, José Beceiro, Luis Escosa, Eloy Muñoz, Iciar Olabarrieta, Francisco Javier Regidor, Miguel Ángel Roa, María del Carmen Viñuela Beneítez, Sara Guillén, Maria Luisa Navarro-Gómez, José Tomás Ramos Amador, on behalf of the Madrid Cohort of HIV-infected mother-infant pairs

**Affiliations:** 1 Hospital Universitario 12 Octubre, Madrid, Spain; 2 Fundación SEIMC-GESIDA, Madrid, Spain; 3 Hospital General Universitario Gregorio Marañón, Instituto de Investigación Sanitaria Gregorio Marañón, (IISGM), CoRISpe, Spain Universidad Complutense, Madrid, Spain; 4 Hospital Clínico San Carlos, Madrid, Spain; 5 Hospital Príncipe de Asturias, Alcalá de Henares, Spain; 6 Servicio de pediatría hospitalaria, enfermedades infecciosas y tropicales, Instituto de Investigación IdiPAZ, Hospital Universitario La Paz, Madrid, Spain; 7 Red de Investigación Translacional en Infectología Pediátrica (RITIP), Madrid, Spain; 8 Hospital General de Móstoles, Móstoles, Spain; 9 Hospital de Getafe, Getafe, Spain; University of Alabama at Birmingham, UNITED STATES

## Abstract

**Background:**

Maternal HIV coinfection is a key factor for mother-to-child transmission (MTCT) of HCV. However, data about HCV MTCT in HIV/HCV-coinfected pregnant women on combined antiretroviral treatment (ART) are scarce. This study assessed the HCV MTCT rate in the Madrid Cohort of HIV-infected women.

**Methods:**

Retrospective study within the Madrid Cohort of HIV-infected pregnant women (2000–2012). Epidemiological, clinical and treatment related variables were analysed for the mother and infant pairs. HCV MTCT rate was determined.

**Results:**

Three hundred thirty-nine HIV/HCV-coinfected women and their exposed infants were recorded. A total of 227 (67%) paired mother-children had available data of HCV follow-up and were included for the analysis. Sixteen children (rate 7.0%, 95%CI 3.7–10.4%) were HCV infected by 18 months of age, none of them coinfected with HIV. HIV/HCV-coinfected pregnant women were mostly of Spanish origin with a background of previous injection drug use. HCV-genotype 1 was predominant. The characteristics of mothers that transmitted HCV were similar to those that did not transmit HCV with respect to sociodemographic and clinical features. A high rate (50%) of preterm deliveries was observed. Infants infected with HCV were similar at birth in weight, length and head circumference than those uninfected.

**Conclusion:**

MTCT rates of HCV among HIV/HCV-coinfected women on ART within the Madrid cohort were lower than previously described. However, rates are still significant and strategies to eliminate any HCV transmission from mother to child are needed.

## Introduction

Hepatitis C virus (HCV) infection has been recognised as a worldwide health problem in both adults and children, being the most common cause of chronic liver disease [1,2]. It is estimated that 5 million children worldwide have an active HCV infection [3].

After the implementation of universal testing of blood transfusion products, mother-to-child transmission (MTCT) became the leading source of HCV infection in children [1,4]. MTCT rates of HCV ranged from 3 to 8% with a weighted rate of transmission of 1.7% when the mother was anti-HCV positive, 4.3% when the mother was positive for HCV RNA, and up to 19.4% when the mother was coinfected with human immunodeficiency virus (HIV)[1,3–7]. The potential biological mechanisms responsible for this association are not yet clearly understood. HIV infection could play a role in the elevation of HCV load facilitating viral transmission, hepatic inflammation, prematurity or severity liver disease [8]. Polis et al. showed in 2007 that maternal HIV/HCV-coinfection increases the MTCT risk of HCV compared with maternal HCV infection alone [9]. More recent studies have confirmed HIV/HCV-coinfection as a potential HCV MTCT risk [6,7,10]. Benova et al. reported in a metaanalysis a HCV MTCT rate of 5.8 (95% CI 4.2–7.8) in monoinfected pregnant women, whereas the transmission rate from HIV/HCV-coinfected pregnant women was 10.8% (7.6–15.2%) [11].

However, many of these studies were performed before the combined antiretroviral therapy (ART) era, when women were more likely to be immunocompromised during pregnancy. Although antiretroviral therapy has no direct effect on HCV replication, the improved immunological condition or other unknown factors might contribute to a reduction of the vertical transmission rate reported in the natural history of the disease.

Fewer studies have examined the rates of MTCT of HCV among HIV-coinfected women with well-controlled HIV disease. In a previous study among HIV/HCV-coinfected mothers from Latin American and the Caribbean, a rate of MTCT of HCV of 8.5% (95% CI, 2.8–21.3) was observed [12]. This rate is similar to the rates of MTCT of HCV observed in multicenter studies conducted among HIV-uninfected women [13, 14].

Therefore, HCV MTCT among HIV/HCV coinfected women on stable antiretroviral treatment may be lower than reported in other coinfected population, presenting current rates of MTCT of HCV that are similar to those monoinfected. The primary objective of this study was to assess the MTCT rate of HCV among HCV/HIV-coinfected women, among infants with follow up testing available, in the ART era in Madrid, Spain.

## Methods

### Design

This was a retrospective study within the Madrid cohort of HIV-infected pregnant women from 2000 to 2012. The Madrid Cohort of HIV-infected mother-infant pairs is a multicenter, prospective and observational study of HIV-1 infected women and their children. Since 2000, mother and infants pairs have been recruited from 8 hospitals in Madrid. The characteristics of the Madrid Cohort have been previously described elsewhere [15]. All HIV/HCV-coinfected pregnant women from the cohort were included in the study (n = 339) and epidemiological, clinical and treatment-related variables were collected during the gestational and delivery period. All children were followed prospectively from birth as part of the Madrid Cohort of mother-infant pairs. Data collection and information available workflow are summarized in **S1 Fig**. A total of 227 (66.8%) paired mother and children with available data from HCV diagnostic tests (serology and molecular) were analysed. Mother-infant pairs without available information (n = 112) about HCV serology or PCR in children were compared with those included in the study (**S1 Table**). In this cohort, HCV PCR was performed per protocol at 3–6 months and HCV serology from 12–18 months of age, simultaneously to HIV serology. Absence of HCV infection was considered as both HCV negative and negative PCR in the first eighteen months. Infants were considered to be HCV-infected if HCV PCR was detected in at least 1 sample and they had persistence of HCV antibodies after 18 months of age. Infants with HCV RNA-positive samples followed by subsequent HCV RNA-negative results at the 6-month visit were classified as having transient HCV infection. Two comparisons were performed: mothers who had transmitted HCV to their infants (n = 16) *versus* mothers who had not (n = 211), and children with HCV infection (n = 16) *versus* non-infected (n = 211).

Written informed consent was obtained for all mother infant pairs. This study was reviewed and approved by the Ethics Committee from Hospital Universitario de Getafe, Madrid.

### Statistical analysis

Chi-squared and Fisher tests were applied to assess differences among the groups for categorical variables. For continuous variables, Student t-test and U-Mann Whitney were applied when appropriate. To estimate the effect of the different sociodemographic, epidemiological and clinical-virological variables, odds ratios (ORs) were calculated and 95% confidence intervals (95% CIs) were assessed using logistic regression. Statistical analysis and graphs were performed using R Software (R Core Team (2018), version 3.5.2, Vienna, Austria. [16].

## Results

### Study population

All HIV/HCV-coinfected mothers from the Madrid cohort of HIV-infected pregnant women were included. A total of 227 paired mother and children were studied. HIV/HCV-coinfected mothers not included in the study due to absence of HCV diagnosis information, had lower HCV VL during pregnancy and were exposed to more rates of ART, compared to the HIV/HCV-coinfected mothers studied **(S1 Table)**.

### Mother’s characteristics

The studied population was mainly from Spain 202/227 (88.9%), Caucasian 206/227 (90.7%), and mostly HIV-infected by parenteral drug use (IVDU) 148/227 (65.2%) and sexual transmission 58/227 (25.5%). Mothers that gave birth to HCV infected children were similar in terms of sociodemographic characteristics, with respect to the non-HCV transmitters. Both groups had similar age at delivery (34 [30–37] *vs*. 34 [31.5–36.5] years) and gestational age (38 [36–38] vs. 37.5 [36.8–39] weeks), with almost 50% of preterm births in both groups **(Table 1)**.

**Table 1:** Characteristics of HIV/HCV-coinfected pregnant women according to the transmission of HCV to their infants.

	Non-HCV transmitters N = 211	HCV transmitters N = 16	*p*-value
**Sociodemographic**			
**Origin**			1.000
Argentina	1 (0.52%)	0 (0.00%)	
Chile	1 (0.52%)	0 (0.00%)	
Colombia	1 (0.52%)	0 (0.00%)	
Russia	2 (1.04%)	0 (0.00%)	
Spain	187 (96.9%)	15 (100%)	
Ukraine	1 (0.52%)	0 (0.00%)	
**Ethnicity**			1.000
Caucasian	191 (98.5%)	15 (100%)	
Native American	3 (1.5%)	0 (0.00%)	
**Route of infection**			1.000
IVDA	136 (68.7%)	12 (75%)	
Sexual	54 (27.3%)	4 (25.0%)	
Transfusion	2 (1.01%)	0 (0.00%)	
Unknown	6 (3.03%)	0 (0.00%)	
**Age at delivery**			0.927
*Years*	34.0 [30.0;37.0]	34.0 [31.5;36.5]	
**Gestational age**			0.386
*Weeks*	38.0 [36.0;38.0]	37.5 [36.8;39.0]	
**CDC classification**			0.277
A	71 (55.9%)	6 (46.1%)	
B	20 (15.7%)	4 (30.8%)	
C	36 (28.3%)	3 (23.1%)	
**Immunologic and Virologic status**			
**CD4 before delivery**			0.142
Count	542 [374;691]	456 [336;477]	
%	31 [21.0;39.6]	31 [30.0;32.0]	
**HIV Viral Load before delivery**			1.000
*Copies/mL*	50.0 [15.0;200]	50.0 [15.0;200]	
**HIV suppressed at delivery *≤50cp/mL***			0.132
No	67 (40.4%)	2 (16.7%)	
Yes	99 (59.6%)	10 (83.3%)	
**HIV Viral Load at delivery**			0.100
*Copies/mL*	50.0 [50.0;200]	50.0 [50.0;50.0]	
**HCV genotype**			0.593
1	55 (55.6%)	6 (75.0%)	
2	7 (7.07%)	0 (0.00%)	
3	19 (19.2%)	0 (0.00%)	
4	18 (18.2%)	2 (25.0%)	
**HCV viral load during pregnancy**			0.154
*Copies/mL*	2·10^5^[15.0;700000]	1·10^6^ [6·10^5^; 4.5·10^6^]	
**Late presenters**			1.000
(diagnosed in the third trimester of pregnancy)			
No	126 (96.9%)	11 (100%)	
Yes	4 (3.08%)	0 (0.00%)	
**Antiretroviral Treatment**			
**ART before pregnancy**			0.521
No	56 (27.2%)	2 (12.5%)	
Yes	146 (70.9%)	13 (81.3%)	
Unknown	4 (1.94%)	1 (6.2%)	
**ART during pregnancy**			0.605
No	15 (7.6%)	0 (0.00%)	
Yes	183 (92.4%)	14 (100%)	
**Time on ART at delivery**			0.148
*years*	5.1 [3.4–7.6]	3.1 [2.6–6.8]	
**Delivery**			
**Type of delivery**			0.801
Caesarean	134 (66.3%)	9 (56.3%)	
Vaginal	68 (33.7%)	7 (43.7%)	
**HIV prophylaxis in delivery:**			0.305
No	16 (7.69%)	2 (12.5%)	
Yes	185 (88.9%)	13 (81.2%)	
Unknown	7 (3.37%)	1 (6.25%)	
**Type of newborn prophylaxis:**			1.000
AZT	183 (98.4%)	13 (100%)	
AZT+3TC+NVP	1 (0.8%)	0 (0.00%)	
AZT+NVP	1 (0.8%)	0 (0.00%)	

**Fig 1:**
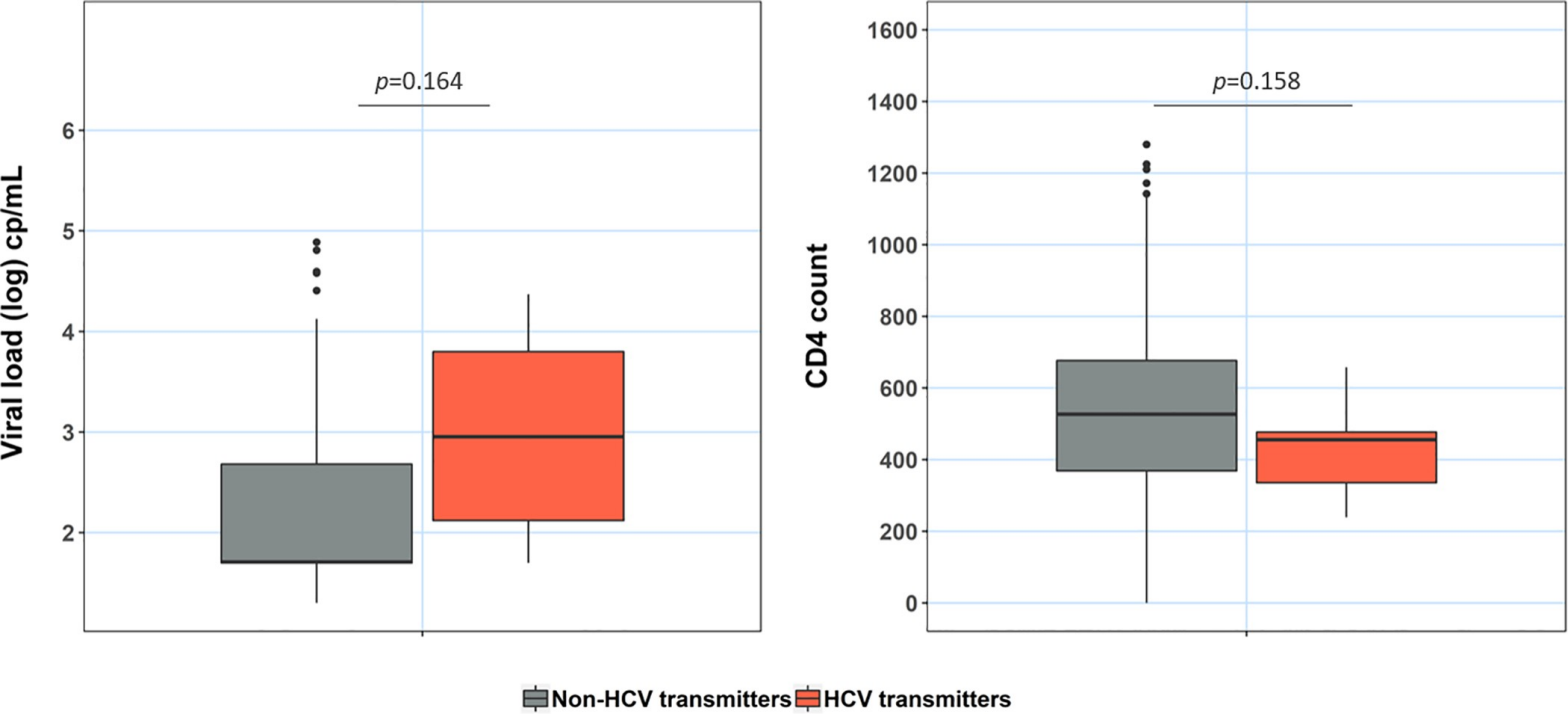
CPW non-transmitters mother (grey); CPWHCV transmitters mother (orange);p:p-value.

Analysing the immunologic and virologic status, HCV transmitters mother presented a slightly lower measurement of CD4 and higher HIV viral load than the non-HCV transmitters mothers. However, these differences did not reach statistical significance (**Fig 1**).

**Fig 2:**
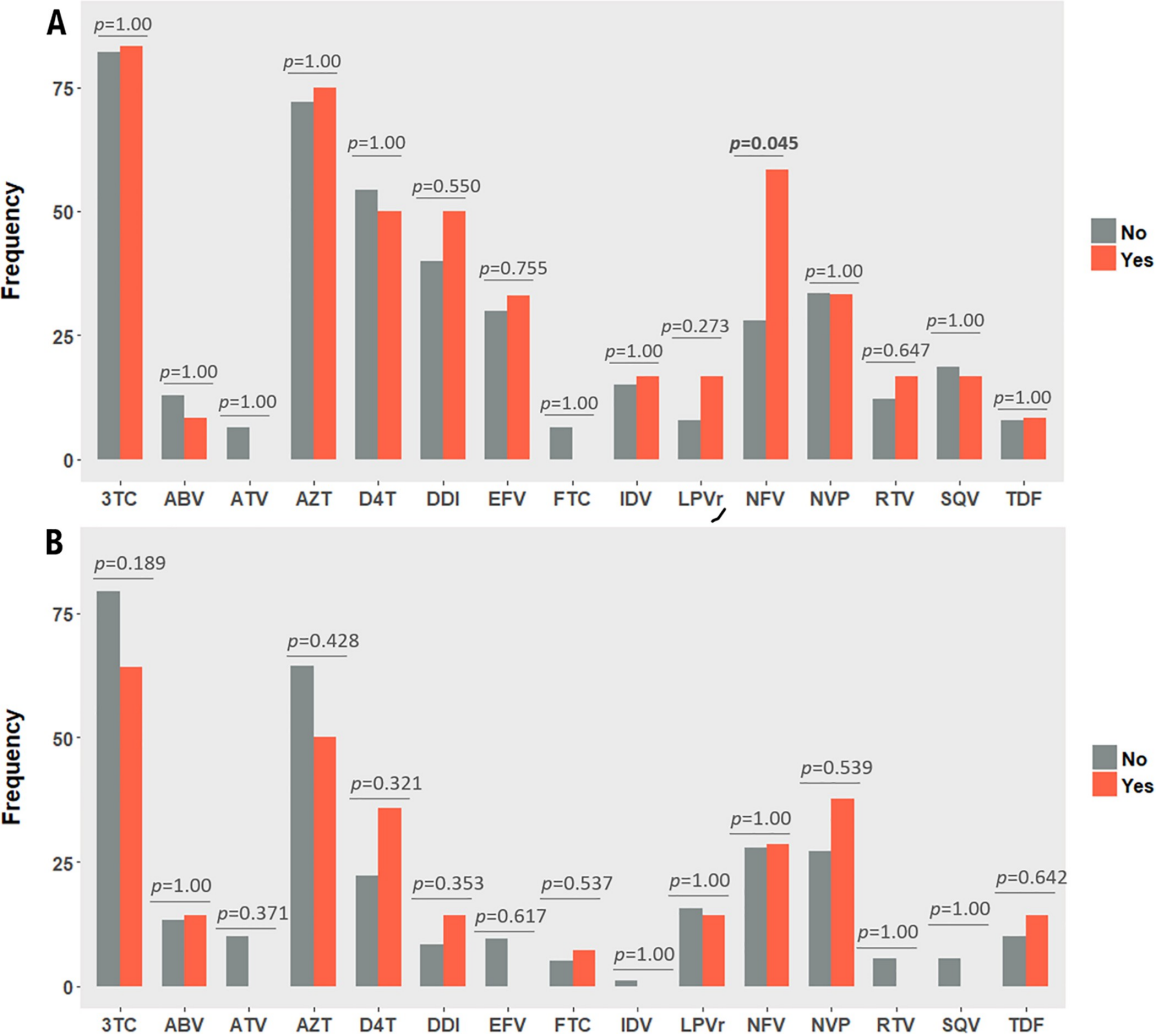
Panel A:ARV drugs before pregnancy;Panel B: ARV drugs during pregnancy.No (grey): CPW non-transmitters mother;Yes(orange):CPW HCVtransmitters mother.3TC:Lamivudine;ABV:Abacavir;ATV:Atazanavir;D4T:Stavudin;DDI:didanosine;EFV:Efavirenz;FTC:Emtricitabine;IDV:Indinavir;LPV/r:Lopinavir/ritonavir;NFV:Nelfinavir;RTV:Ritonavir;SQV:Saquinavir;TDF:Tenofovir;P:p-vale.

In terms of delivery-associated characteristics, there were not consistent differences between HCV transmitters and non-transmitters with regards to the type of delivery or ART **(Fig 2).** HCV transmission was not associated to HIV viral load (OR: 0.8 [0.46;1.2]), last CD4 count before delivery (OR: 1.1 [0.99;1.1]), or vaginal delivery (OR: 1.63 [0.55;4.62]).

### Children characteristics

Among 227 children included in the study, 16 were vertically HCV infected. All of them had a HCV PCR detected in at least 1 sample and a persistence of HCV antibodies after 18 months of age; thus, the HCV transmission rate from HIV/HCV-coinfected women in this cohort was 7.0 (95%CI 3.7–10.4%).

Newborns vertically HCV infected were similar in gender and physical examination (weight, length, and head circumference) at birth with respect to non-HCV newborns (n = 211). No differences were found between groups (**Table 2**).

**Table 2:** Characteristics of newborn infant born to HIV/HCV-coinfected women.

	Non-HCV N = 211	HCV infected N = 16	*p*-value
**Gender**			0.144
Female	94 (44.5%)	5 (31.3%)	
Male	93 (44.1%)	9 (56.2%)	
Unknown	24 (11.4%)	2 (12.5)	
**Weight (newborn)**			0.585
*Grams(g)*	2700 [2348;3028]	2750 [2490;3100]	
**Weight percentile**			0.108
	50.0 [20.5;50.0]	7.00 [7.00;7.00]	
**Length (newborn)**			0.928
*Centimeter (cm)*	47.0 [45.0;49.0]	47.5 [45.6;48.0]	
**Breastfeeding**			1.00
No	173 (97.2%)	11 (100%)	
Yes	5 (2.81%)	0 (0.00%)	

## Discussion

HIV influences the progression of HCV disease, including an increased HCV replication, a decreased rate of HCV clearance during acute infection, and accelerated progression to fibrosis. There is some evidence that HIV viral suppression with ART may reduce the risk of HCV transmission in coinfected mothers [17–19], but the evidence to support this hypothesis is scarce.

Our results showed a 7.0 (95%CI 3.7–10.4%) transmission rate, which appears to be lower than the rate reported in the literature previous to the extended use of ART [11,12]. We also found that mothers who gave birth to HCV infected children were similar with respect to all HIV-1 infection features, including viral load or time on ART at delivery. However, HCV viral load during pregnancy was slightly higher in women who transmitted HCV vertically, although no statistical significance was found, probably due to the low sample size. Same effect could be found in the CD4 count before delivery, where we could observe lower values in transmitters mothers.

Our study does not have the power to answer the question if HCV MTCT was lower in HIV/HCV-coinfected mothers due to improved access to ART, since most women received ART, and no comparisons could be made with HIV/HCV women not on ART. Our results may support the hypothesis of lower rates of HCV transmission in the current era as there is an accompanying lower rate of HIV transmission with better maternal HIV infection control during pregnancy. Although the HCV perinatal transmission rate shown in our study of 7.0% is lower than the previously reported in other studies and in a large meta-analysis [11], the broad confidence intervals overlap.

Among risk factors, the majority of studies with scheduled Caesarean delivery in women with HCV infection, with or without HIV coinfection, have found that the procedure does not reduce the risk of perinatal transmission of HCV [20,21]. These data align well with other studies. Neither, female sex, mode of delivery, nor HCV genotype have been factors with a significant association to HCV coinfection in our population.

There is no consensus on the definition of MTCT of HCV. The definitions of HCV infection (ie viremic or non viremic) and of MTCT differ between studies and this may account for some of the variations between MTCT rates between publications. Nevertheless, it is commonly accepted that MTCT of HCV occurs if there is persistence of anti-HCV antibodies in a child over 18 months of age or the presence of HCV RNA in an infant older than 2 months of age in two different sampling occasions. Since 20% of infants born to HCV infected pregnant mothers may have spontaneous viral clearance, it is important to test for HIV RNA in plasma during the first months of life. Most studies suggest testing for HCV RNA at the age of 2–6 months to early detect HCV infection, along with serum anti-HCV during follow up at 18–24 months in order to verify the persistence or clearance of HCV antibodies [11,12,27]. Since HCV vertical transmission may have a considerable rate of clearance of infection in monoinfected mothers [4] long term follow-up is essential. The proportion of perinatal HCV infected children clearing up the infection has been vaguely studied, and even less data is published in coinfected children, in whom it appear to be much lower [8]. A late paediatric HCV diagnosis, could increase the risk of adverse events, lead to a secondary transmission and result in higher healthcare costs [22,23].

Although our study shows a HCV MTCT lower than the reported in the pre-ART era, it is consistent with current evidence in coinfected ART treated women in Europe and USA [24]. Nevertheless, the transmission rate is still high and strategies to further reduce or eliminate any HCV transmission from mother to child should be implemented. The current potent direct-acting antiviral agents (DAA) against HCV offer new options to eliminate any transmission from mother to children. Ideally, HCV infected women should be treated before gestation. When this is not possible, DAA during pregnancy might be an effective approach given that most vertical transmission occurs at the end of pregnancy or during labour and delivery. Considering that HCV RNA levels decline greatly once treatment is started, most women might achieve undetectable HCV RNA near delivery, a key factor in transmission. So far no DAA has been approved for its use during pregnancy, but sofosbuvir and ledipasvir showed promising safety and PK profiles for its potential use in pregnancy, and animal reproductive toxicity data available is reassuring [24] and initial clinical trials in pregnant women are underway [25].

Preterm deliveries proportion in this study (50%) were higher than the reported in this global same cohort (21.5%) [15], probably due to the high proportion of IDVU mother’s route of infection, but without significant differences between HCV transmitters and non-transmitters. However, higher preterm births in HIV/HCV-coinfected mothers than in HIV monoinfected have been reported [(41.1% vs 15.2%), OR: 3.0 (95% CI 1.6, 5.7)] [26].

Limitations of this study included low sample size and the high percentage of missing diagnosis (30%) in children, which means that a significant number of women with a known HCV infection gave birth to children that remain untested. This fact has already been reported in other cohorts [27,28]. However, when analysing differences between HIV/HCV-coinfected women included and not included within the study, scarce differences were found, avoiding the possible selection bias.

Although multi cohort studies with a higher sample size are needed, this is a wide descriptive group that provides an relevant insight in HCV MTCT among HIV/HCV-coinfected women in the ART era. In conclusion, MTCT rates of HCV among HIV/HCV-coinfected women on ART within the Madrid cohort were lower than previously described. However, rates are still significant and strategies to eliminate any HCV transmission from mother to child are needed.

## Supporting information

S1 Fig (DOCX)

S1 Table Compared mother-infant pairs with and without HCV available information. (DOCX)
