# Supplementary figures and images for: Perinatal HCV Transmission Rate in HIV/HCV Coinfected women with access to ART in Madrid, Spain
Source: PLoS One. 2020 Apr 9;15(4):e0230109. doi: 10.1371/journal.pone.0230109 (PMC7144987; doi:10.1371/journal.pone.0230109)

**Figure S1.**


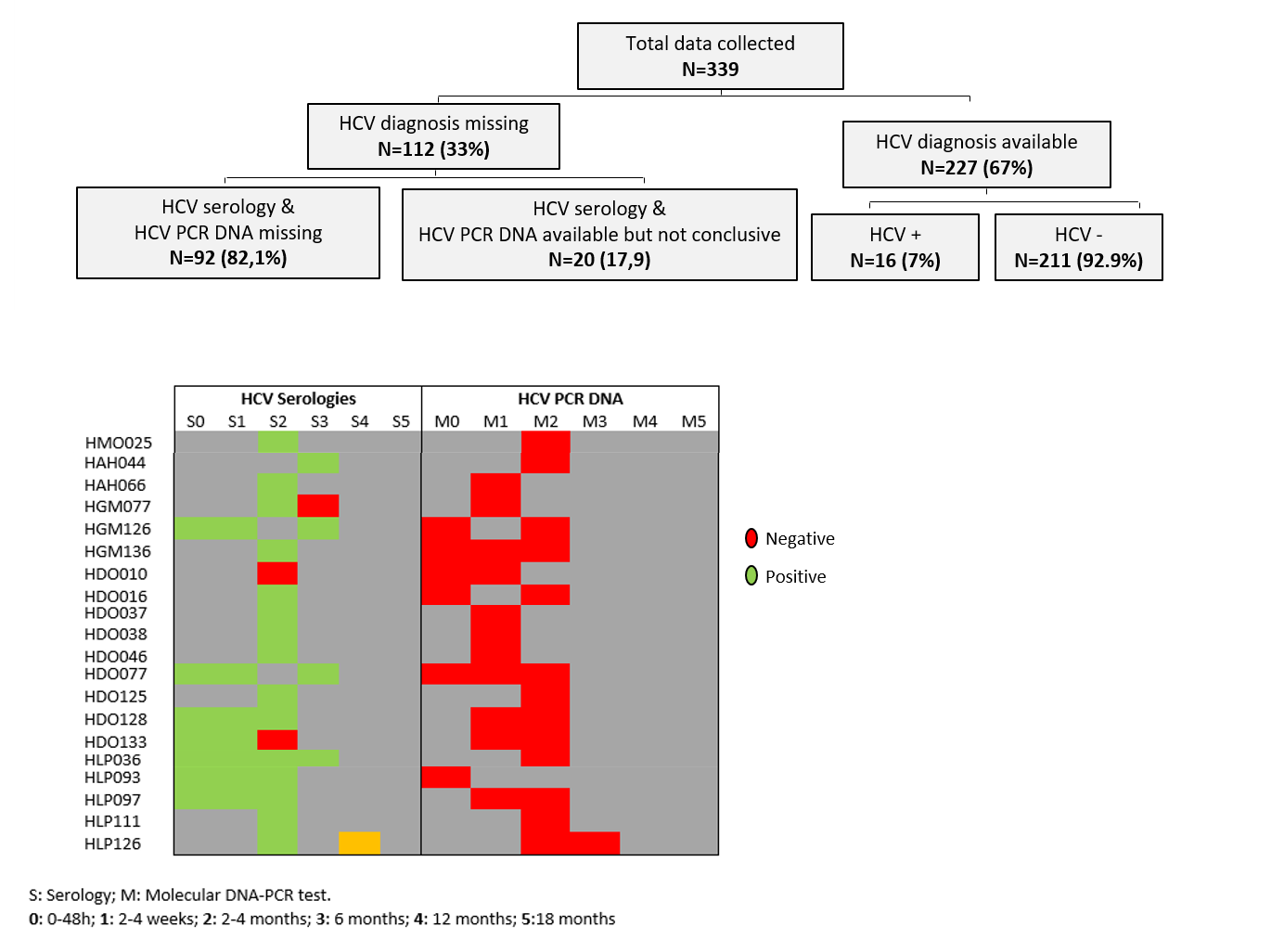

Supplement: S1 Fig — (DOCX) [file pone.0230109.s001.docx]
