# Supplementary material for: Perinatal HCV Transmission Rate in HIV/HCV Coinfected women with access to ART in Madrid, Spain
Source: PLoS One. 2020 Apr 9;15(4):e0230109. doi: 10.1371/journal.pone.0230109 (PMC7144987; doi:10.1371/journal.pone.0230109)
Supplement: S1 Table — (DOCX) [file pone.0230109.s002.docx]

**S1 Table.** Compared mother-infant pairs with and without HCV available information

|  | **Not HCV DX information**  **N=112** | **HCV DX information**  **N=227** | **p.overall** |
| --- | --- | --- | --- |
| **Sociodemographic** |  |  |  |
| **Origin** |  |  | 0.321 |
| Argentina | 0 (0.00%) | 1 (0.48%) |  |
| Chile | 0 (0.00%) | 1 (0.48%) |  |
| Colombia | 0 (0.00%) | 1 (0.48%) |  |
| Equatorial Guinea | 1 (1.00%) | 0 (0.00%) |  |
| Mali | 1 (1.00%) | 0 (0.00%) |  |
| Morocco | 1 (1.00%) | 0 (0.00%) |  |
| Nigeria | 1 (1.00%) | 0 (0.00%) |  |
| Russia | 1 (1.00%) | 2 (0.96%) |  |
| Spain | 95 (95.0%) | 202 (97.1%) |  |
| Ukraine | 0 (0.00%) | 1 (0.48%) |  |
| **Route of infection** |  |  | 0.698 |
| IVDA | 76 (67.9%) | 148 (65.2%) |  |
| Sexual | 28 (25.9%) | 58 (27.0%) |  |
| Transfusion | 1 (0.93%) | 2 (0.93%) |  |
| Unknown | 3 (2.78%) | 6 (2.79%) |  |
| **Age at delivery** |  |  | 0.752 |
| *years* | 33.0 [31.0;37.0] | 34.0 [30.2;37.0] |  |
| **Gestational age** |  |  | 0.391 |
| *weeks* | 37.0 [35.2;38.0] | 38.0 [36.0;38.0] |  |
| **CDC classification** |  |  | 0.818 |
| A | 48 (59.2%) | 96 (56.2%) |  |
| B | 18 (22.2%) | 31 (18.1%) |  |
| C | 15 (18.5%) | 44 (25.7%) |  |
|  | |  |  |
| **CD4 before delivery** |  |  |  |
| count | 460 [338;818] | 520 [371;672] | 0.855 |
| % | 27.0 [22.0;35.0] | 31.0 [21.0;39.2] | 0.328 |
| **HIV Viral Load before delivery** |  |  |  |
| *Copies/mL* | 232 [50.0;7136] | 275 [50.0;4515] | 0.870 |
| **HIV Viral Load at delivery** |  |  |  |
| *Copies/mL* | 50.0 [50.0;490] | 50.0 [50.0;190] | 0.492 |
| **HIV suppression at delivery**  **<50 copies/mL** |  |  | **0.891** |
| No | 32 (40%) | 69 (38.8%) |  |
| Yes | 48 (60%) | 109 (61.2%) |  |
| **HCV genotype** |  |  | 0.917 |
| 1 | 29 (60.4%) | 61 (57.0%) |  |
| 2 | 2 (4.17%) | 7 (6.54%) |  |
| 3 | 7 (14.6%) | 19 (17.8%) |  |
| 4 | 10 (20.8%) | 20 (18.7%) |  |
| **HCV viral load during pregnancy (n=21)** |  |  |  |
| Log *Copies/mL* | 7.1 [5.3;7.2] | 5.3 [1.2;6] | 0.056 |
|  |  |  |  |
| **Antiretroviral Treatment** |  |  |  |
| **ART before pregnancy** |  |  | 0.114 |
| No | 40 (37.0%) | 58 (26.2%) |  |
| Yes | 66 (61.1%) | 159 (71.9%) |  |
| Unknown | 2 (1.85%) | 4 (1.81%) |  |
| **ART during pregnancy** |  |  | 0.065 |
| No | 14 (14.4%) | 15 (7.08%) |  |
| Yes | 83 (85.6%) | 197 (92.9%) |  |
| **Time on ART at delivery** |  |  | 0.632 |
| *Years* | 5.2 [3.0;18.0] | 4.9 [3.3;13.1] |  |
|  |  |  |  |
|  |  |  |  |
| **Delivery** |  |  |  |
| **Type of delivery** |  |  | 0.769 |
| Caesarean | 76 (69.1%) | 151 (66.8%) |  |
| Vaginal normal | 34 (30.9%) | 75 (33.2%) |  |
| **HIV prophylaxis at delivery:** |  |  | 0.921 |
| No | 2 (1.8%) | 1 (0.4%) |  |
| Yes | 97 (85.8%) | 198 (87.2%) |  |
| Unknown | 14 (12.4%) | 28 (12.3%) |  |
| **Type of newborn prophylaxis:** |  |  | 1.00 |
| AZT | 95 (98.9) | 196 (99%) |  |
| AZT+3TC+NVP | 0 | 1 (0.5%) |  |
| AZT+NVP | 1 (1.1%) | 1 (0.5%) |  |
